# Single-Cell RNA Sequencing Reveals Heterogeneity in the Tumor Microenvironment between Young-Onset and Old-Onset Colorectal Cancer

**DOI:** 10.3390/biom12121860

**Published:** 2022-12-12

**Authors:** Gui-Ming Li, Guo-Zhong Xiao, Peng-Fei Qin, Xing-Yang Wan, Yuan-Ji Fu, Yi-Hui Zheng, Min-Yi Luo, Dong-Lin Ren, Shi-Ping Liu, Hua-Xian Chen, Hong-Cheng Lin

**Affiliations:** 1 Department of Colorectal Surgery, The Sixth Affiliated Hospital, Sun Yat-sen University, Guangzhou 510655, China; 2 Guangdong Provincial Key Laboratory of Colorectal and Pelvic Floor Diseases, The Sixth Affiliated Hospital, Sun Yat-sen University, Guangzhou 510655, China; 3 Guangdong Institute of Gastroenterology, Guangzhou 510655, China; 4 BGI-Shenzhen, Beishan Industrial Zone, Shenzhen 518083, China; 5 BGI Education Center, University of Chinese Academy of Sciences, Shenzhen 518083, China; 6 Shenzhen Key Laboratory of Single-Cell Omics, BGI-Shenzhen, Shenzhen 518083, China; 7 Department of Gastrointestinal Surgery, The Sixth Affiliated Hospital, Sun Yat-sen University, Guangzhou 510655, China

**Keywords:** single-cell RNA sequencing, colorectal cancer, tumor microenvironment, age

## Abstract

Background: The incidence of sporadic young-onset colorectal cancer (yCRC) is increasing. Compared with old-onset colorectal cancer (oCRC), yCRC has different clinical and molecular characteristics. However, the difference in the tumor microenvironment (TME) between yCRC and oCRC remains unclear. Methods: Fourteen untreated CRC tumor samples were subjected to single-cell RNA sequencing analysis. Results: B cells and naïve T cells are enriched in yCRC, while effector T cells and plasma cells are enriched in oCRC. Effector T cells of yCRC show decreased interferon-gamma response and proliferative activity; meanwhile, Treg cells in yCRC show stronger oxidative phosphorylation and TGF-β signaling than that in oCRC. The down-regulated immune response of T cells in yCRC may be regulated by immune and malignant cells, as we observed a downregulation of antigen presentation and immune activations in B cells, dendritic cells, and macrophages. Finally, we identified malignant cells in yCRC and oCRC with high heterogeneity and revealed their interactions with immune cells in the TME. Conclusions: Our data reveal significant differences of TME between yCRC and oCRC, of which the TME of yCRC is more immunosuppressive than oCRC. Malignant cells play an essential role in the formation of the suppressive tumor immune microenvironment.

## 1. Introduction

Colorectal cancer (CRC) is the third most common cancer in the worldwide [1]. It is commonly considered as a disease of older people. Only a few cases occur in young adults [2]. Although effective screening has reduced the mortality of CRC in recent decades, morbidity of sporadic young-onset CRC (yCRC) is continuously increasing [3]. In recent years, more and more studies have reported that the clinicopathological and molecular characteristics of yCRC differ from those of old-onset colorectal cancer (oCRC) [4,5]. Patients with yCRC often present with mucinous and signet-ring features, poorly differentiated histology, and more advanced disease; hence, yCRC often shows a more aggressive and poorer prognosis [6]. Furthermore, a recent study highlighted that gut microbial diversity is increased in patients with yCRC [7]. A recent study also highlighted that the immune cell infiltration in CRC tissues showed differential heterogeneity patterns among different age groups [8]. However, the differences in the tumor microenvironment (TME) and molecular characterization between yCRC and oCRC remain unclear, making it difficult to examine the differences in their pathogenesis.

In recent years, chemotherapy, radiotherapy, and immunotherapy have played an increasingly important role in the treatment of CRC. Accumulating evidence shows that the TME influences tumor progress and response to therapy [9,10]. The aged TME can contribute to the development of chemotherapy resistance [11]; this may be related to the fact that fibroblasts and immune cells in the TME are susceptible to age-related effects [11]. Moreover, recent studies in patients with metastatic melanoma reported that patients aged 60 years or older responded more efficiently to anti-PD1 and the likelihood of response to anti-PD1 treatment increased with age [12,13]. As these data indicate that the age-related differences in the TME are associated with variations in treatment responses; therefore, the development of an age-specific treatment may be warranted. Furthermore, few studies have directly investigated the possible significant differences in the TME between younger and older patients. Single-cell RNA sequencing (scRNA-seq) has revealed the highly complex cellular composition in the TME with high resolution. It is a powerful tool for analyzing the cellular components and their interactions in the tumor ecosystem [14,15]. However, a comprehensive description of the TME in yCRC and oCRC at the single-cell resolution level is lacking. Thus, the study of age-mediated differences in the TME of CRC at the single-cell level may help in the development of more effective strategies for CRC.

Herein, scRNA-seq analysis was performed on 14 patients with untreated CRC, including 7 yCRC and 7 oCRC. We analyzed the TME heterogeneity between the two groups, and then further analyzed the T-cell, B-cell, and myeloid cell subsets and their interactions. Our data showed TME differences between yCRC and oCRC. Hopefully, our findings may guide the development of effective therapies to benefit a wide range of patients.

## 2. Materials and Methods

### 2.1. Human Sample Collection

This study was approved by the Ethics Committee of the Sixth Hospital of Sun Yat-sen University (no. 2022ZSLYEC-581). We collected tumor tissues from 14 patients who were pathologically diagnosed with CRC, including 7 yCRC (age <50 years) and 7 oCRC (age ≥50 years). All patients were diagnosed with primary tumors and untreated. The patients’ clinical characteristics including age, gender, pathological subtype, and tumor stage are listed in Supplementary Table S1.

### 2.2. Single-Cell Isolation

Fresh tumor tissue samples were washed with DPBS (Hyclone, Logan, UT, USA) and cut into 2–4 mm^3^ slices. The tissue samples were suspended in 1640 medium (Gibco Tour, Grand Island, NY, USA) and transferred to a gentleMACS C tube containing an enzyme mix. This tube was connected to the gentleMACS Octo Dissociator, and the appropriate procedure was performed. The digested tissue was then passed through a 70-μm strainer, and the cell suspension was centrifuged at 300 g for 7 min. After removal of the supernatant, the cells were subsequently resuspended in erythrocyte lysis buffer and incubated at 4 °C for 10 min to remove the erythrocytes. The cells were centrifuged at 300 g for 10 min at 4 °C, and the supernatant was removed without disturbing the cell precipitation. Finally, 5 mL of frozen wash buffer (0.04% BSA(Sangon Biotech, Shanghai, China) in DPBS) was added to the cells and the mixture was stirred with gentle puffing to resuspend the cell precipitate. The cell viability in all samples was >80% and determined via trypan blue (Sigma, Darmstadt, Germany) staining. The final cell concentration was adjusted to 700–1200 cells/μL for single-cell library preparation.

### 2.3. scRNA-Seq

Gene expression libraries were constructed using the DNBelab Single-Cell Kit (MGI, Shenzhen, China) following the manufacturer’s instructions. scRNA-seq was then performed using the DNBelab C4 platform. Briefly, single-cell suspensions were used for droplet generation, lactation, bead collection, reverse transcription, and DNA amplification to generate barcode libraries. Indexed scRNA-seq libraries were constructed according to the manufacturer’s protocol. The sequencing libraries were quantified using the QubitTM ssDNA Assay Kit (Thermo Fisher Scientific, Waltham, MA, USA, #Q10212).

### 2.4. Single-Cell Gene Expression Matrix Acquisition and Cell Clustering

Raw sequencing reads were filtered and demultiplexed using PISA (https://github.com/shiquan/PISA (accessed on 20 August 2021)). The filtered reads were aligned to the hg38 genome. The cell-gene count matrix was generated using PISA. The Seurat R package (https://github.com/satijalab/seurat (version 4.1.0)) (accessed on 20 August 2021) [16] was used to process the messages in R (https://www.r-project.org/ (version 4.1.2)) (accessed on 20 August 2021). High-quality cells were selected for preservation based on the following criteria: 1) the number of genes identified in individual cells ranged from 300 to 6000, and 2) the percentage of mitochondrial gene expression in individual cells, an indicator of apoptosis-related cellular status, was <20%. As a result, 46,155 cells (yCRC) and 38,481 cells (oCRC) were included in the following analysis. The gene expression data were normalized using the Seurat package and the normalization method “LogNormalize” to reduce the number of discrete gene expression counts. Then, the Seurat “FindVariableFeatures” function was used to generate highly variable genes (HVGs), select the top 2000 HVGs, perform a principal component analysis, and select the top 15 significant principal components to perform a unified flow approximation and projection (UMAP) dimensionality reduction [17]. Harmony was used to remove the batch effects between samples for clustering, and the parameter “group.by.vars” was set to “sample” The cells were clustered using the “FindClusters” function (resolution = 0.8) and visualized in two dimensions using UMAP.

### 2.5. Identifying Marker Genes and Differentially Expressed Genes

We used Seurat’s “FindAllMarkers” function to identify marker genes for each cluster. Marker genes identified using the “FindAllMarkers” function were required to have an average expression: a cluster with an avg_logFC of >0.25-fold higher than the average expression in the other clusters and a detectable expression in >25% of cells from that cluster. We calculated the number of differentially expressed genes (DEGs) in cell subpopulations using Seurat’s “Findmarker” function and using the Wilcoxon rank-sum test based on default parameters. |avg_log2FC| ≥ 0.25 and *p*_val < 0.05 were assigned as the cutoff criteria.

### 2.6. Cell Developmental Trajectory

Trajectory analysis was performed using the Monocle 3 package [18] (http://cole-trapnell-lab.github.io/monocle3/ (version 1.2.9)) (accessed on 20 August 2021) in RStudio. Data pre-processing was performed using the “preprocess_cds” function, with the number of dimensions set at 50. Dimensionality reduction and clustering were performed using the “reduce_dimension” and “cluster_cells” functions, respectively. To obtain consistent UMAPs across these tools, custom scripts were used to transfer the UMAP calculated in “Seurat” to “Monocle3” before running the main functions “learn_graph” and “order_cells” to determine the trajectory and pseudotime. Then, a principal graph was fitted within each cluster using the “learn_graph” function, and the pseudotime order of cells was visualized using the “plot_cells” functions, as appropriate with the pseudotime coloring option. After constructing the cell trajectories, the DEGs along the pseudotime were detected using the “graph_test” function.

### 2.7. Pathway Enrichment Analysis

The ClusterProfiler [19] R package (https://www.bioconductor.org/packages/clusterProfiler (version 4.2.2)) (accessed on 20 August 2021) was used to perform Gene Ontology (GO) analyses. Gene sets with a *p*-value of <0.05 were considered to be significantly enriched. The R package GSVA [20] (https://bioconductor.org/packages/GSVA (version 1.42.0)) (accessed on 20 August 2021) was used to perform the gene set variation analysis (GSVA). The relevant pathways were selected from 50 hallmark pathways described in the molecular signature database. The differential activated pathways between cells from yCRC and oCRC tissues were tested using the generalized linear modeling feature of the Limma package (version 3.50.1). Pathways with an adjusted *p*-value of <0.05 were considered to be significantly differentially activated.

### 2.8. The Cancer Genome Atlas Data Analysis

The colon adenocarcinoma and rectal adenocarcinoma data obtained from The Cancer Genome Atlas (TCGA) database were used to confirm the difference in immune cell infiltration rate between yCRC and oCRC and for survival analysis. The gene expression data and clinical phenotype data were downloaded from UCSC Xena (http://xena.ucsc.edu/ (accessed on 20 July 2022)). TCGA data were used to evaluate the prognostic effect of gene sets derived from specific cell clusters. To assign “gene signature scores” to individual cells, GSVA was carried out using standard settings. The following plasma cell marker genes were used in the GSVA: *IGLC2*, *IGLC3*, *IGHG4*, *IGHG3*, *IGHA2*, *IGHG1*, *IGKC*, *CD138*, *CD38*, *IGHA1*, *JCHAIN*, and *IGLC1*. Then, the patients were divided into high- and low-expression groups using the “surv_cutpoint” function and identified the optimal cutoff value. The survival curves were plotted using the ggsurvplot function.

### 2.9. InferCNV Analysis to Identify Malignant Cells

The copy number variation (CNV) score in the 14 patients with epithelial cells was calculated based on the single-cell transcriptomic profiles using InferCNV [21] (https://github.com/broadinstitute/inferCNV (version 1.10.1)) (accessed on 20 August 2021). Stromal cells including fibroblasts and endothelial cells were selected as references. For the inferCNV analysis, the following parameters were used: “denoise,” default hidden markov model settings, and a value of 0.1 as the “cutoff” value. Finally, the subclusters with relatively higher CNV scores were considered malignant cells. A total of 5918 epithelial cells were identified as normal epithelial cells, while 17,833 epithelial cells were considered malignant cells.

### 2.10. Cell-Cell Communication Analysis

The CellChat algorithm (https://github.com/sqjin/CellChat (version 1.1.3) (accessed on 20 August 2021) was used to infer the cell–cell interactions [22]. Briefly, we followed the official workflow and loaded the normalized count from Seurat to CellChat for processing. The ligand-receptor database contained in CellChat was used to infer intercellular communication. The CellChat algorithm was then run to calculate the probable interactions and pathways via the “computeCommunProb” and “computeCommunProbPathway”. We also ran the “filterCommunication” to filter out interactions with less than 10 cells in each cell type.

### 2.11. Statistical Analysis

The cell distribution was compared between the two groups of cells using unpaired two-tailed Wilcoxon rank-sum tests. The gene expression or gene signature was compared between the two groups of cells using an unpaired two-tailed Student’s *t*-test. Pathway activity correlation using Spearman’s correlation test. Statistical significance was set at a *p*-value of <0.05. Significance levels are indicated as * *p* < 0.05, ** *p* < 0.01 and *** *p* < 0.001.

## 3. Results

### 3.1. Single-Cell Transcriptomic Profiling of yCRC and oCRC

**Figure 1:**
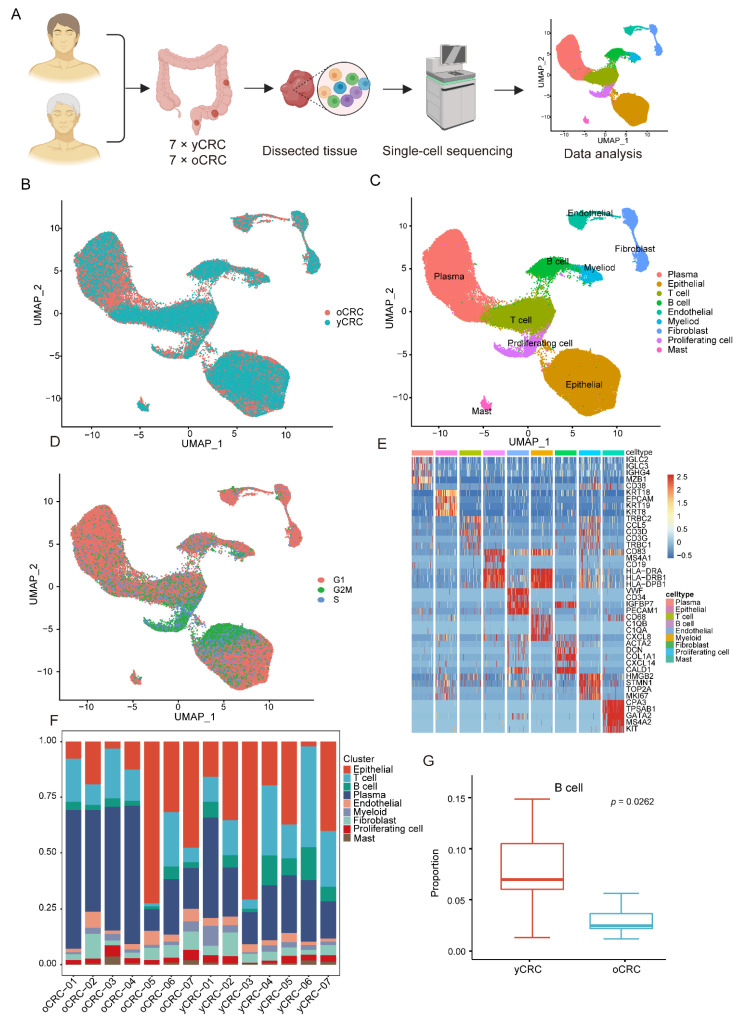
Overview of ScRNA-seq analysis of yCRC and oCRC. (**A**) Schematic diagram of the experimental design and workflow. (**B**) The UMAP plot showing the cells in yCRC and oCRC, different color represents different groups. (**C**) The UMAP plot showing the cell types in yCRC and oCRC, different color represents different cell types. (**D**) The UMAP plot showing the cells in yCRC and oCRC, different color represents different cell cycle phases. (**E**) Heatmap showing differential expression of marker genes in each cell type. (**F**) The bar chart showing the proportion of cell types in different patients. (**G**) Comparison of B cells proportions between yCRC and oCRC.

To characterize the TME of CRC in different age groups, tumor tissues were collected from 14 patients with untreated CRC (7 yCRC and 7 oCRC) for scRNA-seq (Figure 1A). The clinical and pathological information of the patients, including age, gender, and tumor TNM stage, are presented in Supplementary Table S1. After strict quality control and filtration, 84,636 cells were used for subsequent analysis. A total of 46,155 cells were obtained from patients with yCRC, while 38,481 cells were obtained from patients with oCRC (Figure 1B). The quality control criteria and filtering steps are presented in the Section 2. After eliminating the batch effects between multiple samples, Seurat was used for cell classification and marker gene identification. We identified and visualized 23 cell clusters using UMAP dimensionality reduction method (Figure S1A). According to the classical cell markers reported in previous studies [23,24,25,26], we identified the cell type of each cluster (Figure 1C and Figure S1B). Non-immune cells were mainly composed of epithelial cells (*EPCAM* and *KRT19*), fibroblasts (*ACTA2* and *DCN*), and endothelial cells (*CD34* and *VWF*). The identified immune cells include T cells (*CD3D* and *CD3G*), B cells (*CD19* and *MS4A1*), plasma cells (*MZB1* and *CD38*), myeloid cells (*CD68* and *C1QB*), and mast cells (*MS4A2* and *KIT*). None of these cell types were group- or patient-specific. We noticed a group of cells that highly expressed *MKI67* and *TOPA2*, and annotated them as proliferating cells. By evaluating the cell cycle, we observed that this group of proliferating cells was primarily in the G2M phase of the cell cycle (Figure 1D), thus confirming their active proliferation status. We used the FindAllmarkers function to explore the marker genes for each cell type. The marker genes for each cell type were visualized as a heatmap (Figure 1E). All cell types had unique gene expression patterns, demonstrating the reliability of our clustering and annotation results.

Furthermore, we elucidated the proportions of cell types in different groups and in each patient (Figure 1F and Figure S1C). All cell subsets were retrieved in different patients including those with yCRC and oCRC. The proportions of all cell types in yCRC and oCRC tissues showed a similar distribution. However, the B cells differed significantly between the two groups (Figure 1G). In addition, plasma cells and fibroblasts were mainly from oCRC, while T cells, myeloid cells, and endothelial cells were mainly from yCRC (Figure S1C). Collectively, our data reveal the TME heterogeneity between yCRC and oCRC and highlight the significant differences in tumor-infiltration immune cells between the two groups.

### 3.2. Features and Heterogeneity of T-Cell Subtypes in yCRC and oCRC

**Figure 2:**
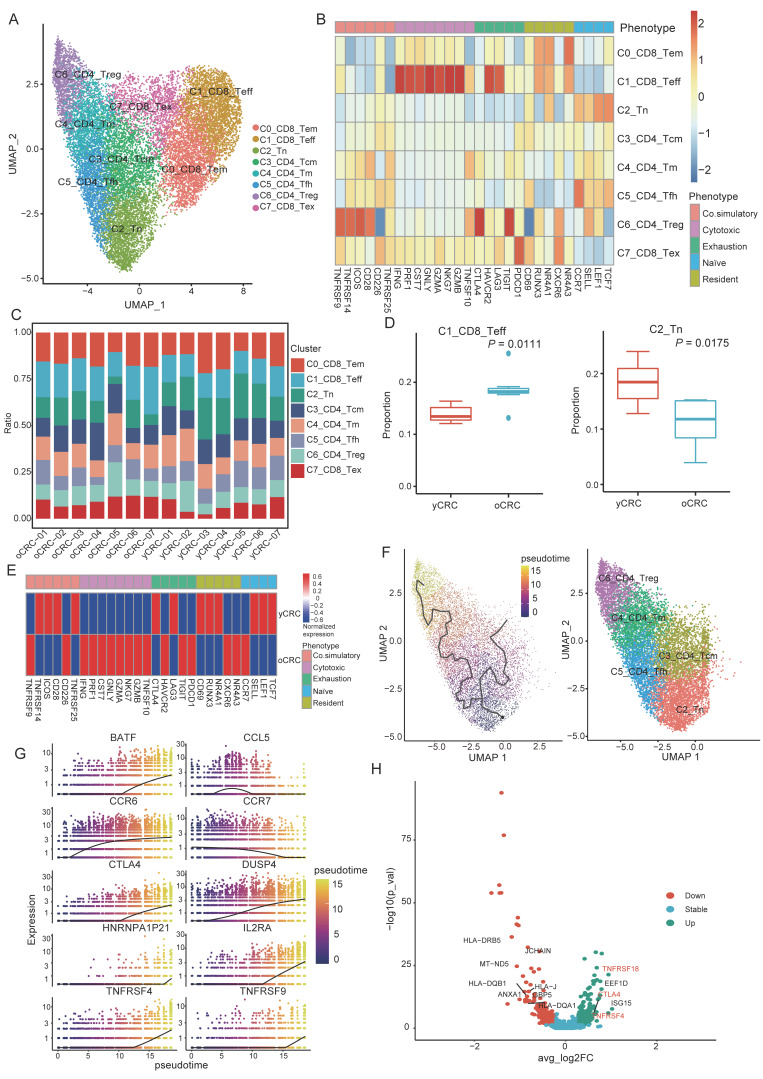
Features and heterogeneity of T cell subtypes in yCRC and oCRC. (**A**) UMAP plot showing the subgroups of T cells. (**B**) Heatmap showing expression of T cell functional phenotype genes in different T cell subtypes. (**C**) The bar chart showing the proportion of T cell subtypes in different patients. (**D**) Comparison of selected T cell subtype populations between yCRC and oCRC. (**E**) Heatmap showing expression of T cell functional phenotype genes in different groups. (**F**) Pseudotime reconstruction and developmental trajectory of CD4+T cells inferred by Monocle 3. Cells are colored by pseudotime (left). UMAP visualization of CD4+T cells (right). (**G**) Differential genes expression dynamics resolved along pseudotime. (**H**) Volcano plot showing differentially expressed genes between C6_CD4_Treg cells of yCRC and oCRC. yCRC versus oCRC, down (up) represents differential genes downregulated (upregulated) in yCRC C6_CD4_Treg.

T cells are the primary immune cell type that regulates antitumor immunity, and we subclustered and unsupervised reclustered T cells, revealing eight clusters of T cells (Figure 2A). We divided them into three CD8+ T-cell clusters with expression of *CD8A*, including effector memory T cells C0_CD8_Tem (expressing *GZMK* and *NKG7* effector T-cell markers, while also highly expressing some T-cell tissue-resident genes such as *RUNX3 and NR4A1*), effector T cells C1_CD8_Teff (highly expressing the cytotoxic genes *GZMB*, *GZMA*, *NKG7*, and *IFNG*) and exhausted T cells C7_CD8_Tex (expressing *PDCD1*, and *LAG3*) (Figure 2B and Figure S2A). The CD4+ T-cell clusters included central memory T cells C3_CD4_Tcm (*IL7R+ANXA1+*), memory T cells C4_CD4_Tm (*CD44+S100A4+GPR183+*), follicular helper T cells C5_CD4_Tfh (*CXCR5+BCL6+*), and regulatory T cells (Treg) C6_CD4_Tregs (*FOXP3+TNFRSF4+*) (Figure S2A). Other cells expressing the marker of naïve T cells, such as *CCR7*, *SELL*, *LEF1*, and *TCF7*, were defined as naïve T cells (C2_Tn) (Figure 2B).

Each cell subtype was distributed among the yCRC and oCRC groups (Figure 2C). The proportion of C2_Tn was higher in the yCRC group than that in the oCRC group, while a higher proportion of C1_CD8_Teff was observed in oCRC (Figure 2C,D). In addition, we compared the gene expression levels associated with T-cell functional phenotypes between the yCRC and oCRC groups (Figure 2E). Cytotoxic genes were highly expressed in the oCRC group, whereas naïve T-cell genes were highly expressed in the yCRC group. Moreover, the yCRC group expressed higher levels of co-stimulatory-related molecules (Figure 2E), which may imply that Treg cells have a more potent immunosuppressive function in yCRC.

**Figure 3:**
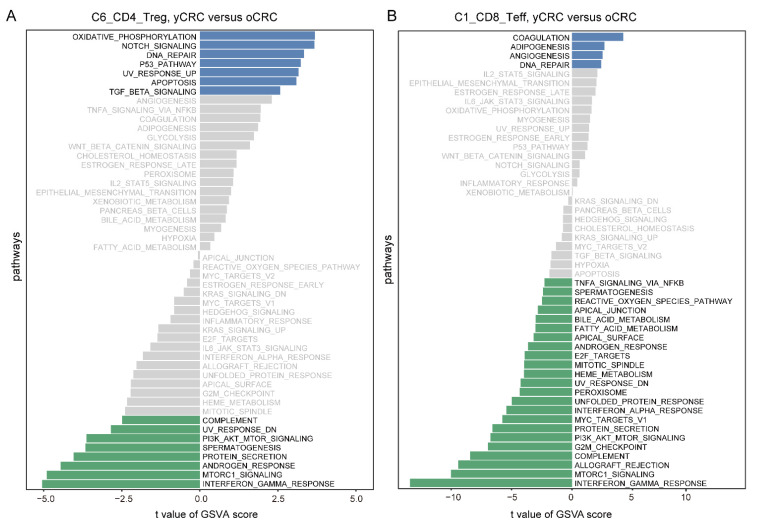
Pathway differences between Treg cells and effector T cells between yCRC and oCRC. (**A**) Differences in Hallmark pathways activities scored per cell by GSVA between yCRC and oCRC C6_CD4_Treg cells. t values are from linear models, corrected for effects from the patient of origin. (**B**) Differences in Hallmark pathways activities scored per cell by GSVA between yCRC and oCRC C1_CD8_Teff cells. t values are from linear models, corrected for effects from the patient of origin.

We used monocle3 to elucidate the developmental trajectory of CD4+ T cells. Trajectory analysis showed that C2_Tn was at the beginning of the developmental trajectory and C6_CD4_Treg was at the end of the developmental trajectory (Figure 2F), confirming the reliability of our cell annotation results. The expression of naïve genes (*CCR7*) decreased, while that of exhausted genes (*CTLA4*) increased as the developmental time progressed (Figure 2G). Furthermore, the expression of co-stimulatory molecules (*TNFRSF4* and *TNFRSF9*) increased during pseudotime (Figure 2G). We further confirmed the potential functional differences between yCRC and oCRC Treg cells. We compared the gene expression profiles of the two groups of C6_CD4_Treg cells (Figure 2H). The expression of co-stimulatory molecules (*TNFRSF4* and *TNFRSF18*) was higher in yCRC C6_CD4_Treg cells. These co-stimulatory molecules enhance the proliferation and activation of Treg cells and conventional T cells. *CTLA4* also showed higher expression in the yCRC group (Figure 2H). To explore their potential pathway differences, we performed a GSVA of C6_CD4_Treg cells from the yCRC and oCRC groups. The yCRC C6_CD4_Treg cells showed stronger oxidative phosphorylation and TGF-β signaling (Figure 3A). Previous studies have shown that Treg cells undergo metabolic reprogramming in tumors. By promoting oxidative phosphorylation and inhibiting glycolysis, the cells adapt to a low-sugar and high-lactate TME [27], thereby promoting Treg proliferation, differentiation, and immunosuppressive functions. The TGF-β signaling pathway plays a key role in the suppression of antitumor immune response by Treg cells, and blockage of the TGF-β signaling pathway can potentially weaken the immunosuppressive function of Treg cells to enhance the antitumor response [28]. All of these results may suggest that C6_CD4_Treg cells in yCRC showed stronger immunosuppressive activity.

Furthermore, we evaluated the effector T cells. GSVA results showed that adipogenesis and angiogenesis were enriched in yCRC. Previous studies have shown that adipogenesis and angiogenesis pathways may be associated with poor prognosis in patients with tumors [29,30]. By contrast, the effector T cells in yCRC showed lower cell proliferation-related pathway G2M/E2F and interferon-gamma pathway activities (Figure 3B). The interferon gamma pathway plays an important role in the proliferation of effector T cells [31,32]. Consistent with this result, the cell proliferation pathway scores were lower in the yCRC group (Figure S3A). These results suggest that the effector T cells in patients with yCRC may be in a relatively resting phase, thus showing lower proliferative activity and cytotoxic function.

In conclusion, yCRC has a higher abundance of naïve T cells compared with oCRC, while effector T cells are significantly enriched in oCRC. The Treg cells of yCRC have higher immunosuppressive activity. In addition, the effector T cells from yCRC have lower proliferative activity and cytotoxicity compared with those from oCRC.

### 3.3. Heterogeneity of B Cells and Plasma Cells in the TME of yCRC and oCRC

**Figure 4:**
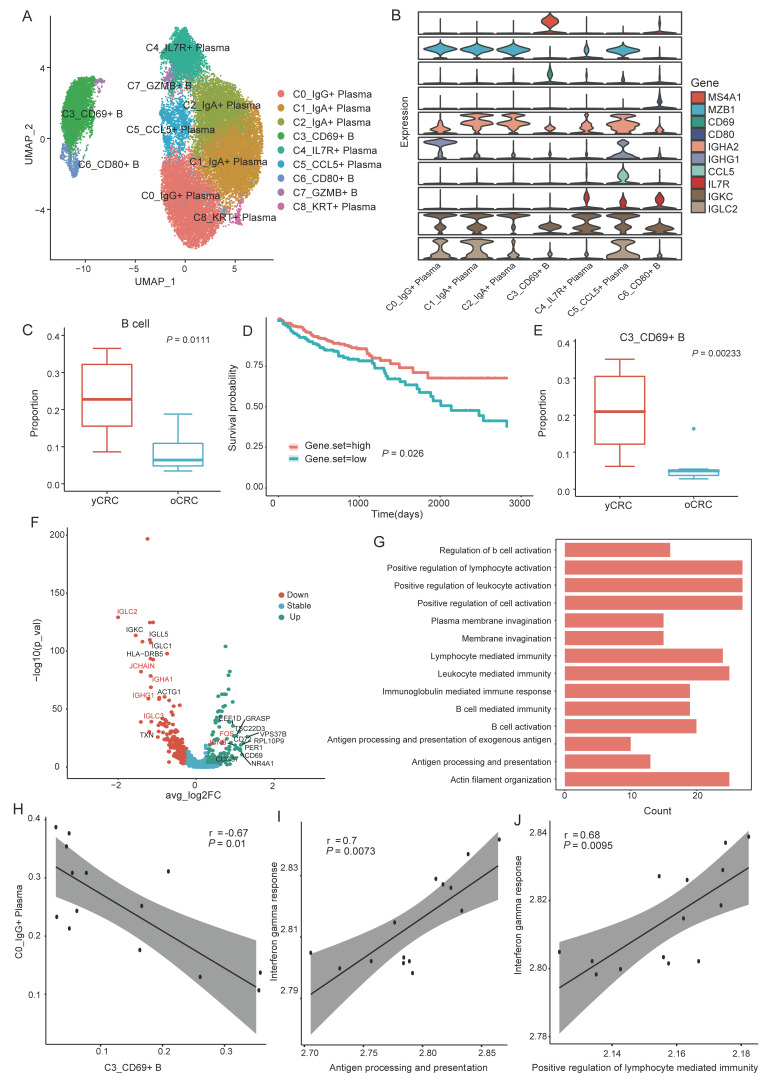
B cells and plasma cells subtypes and their heterogeneity in yCRC and oCRC. (**A**) UMAP plot showing the subtypes of B/plasma cells. (**B**) Violin plots comparing the relative expression of marker genes across various B/plasma subtypes. (**C**) Comparison of the B cell proportion between yCRC and oCRC. (**D**) The Kaplan–Meier overall survival curves of TCGA COAD and READ patients grouped by different expression levels of signature genes of plasma cells marker. Set of plasma cell marker genes: IGLC2, IGLC3, IGHG4, IGHG3, IGHA2, IGHG1, IGKC, CD138, CD38, IGHA1, JCHAIN, and IGLC1. (**E**) The comparisons of C3_CD69+B cells proportion between yCRC and oCRC. (**F**) Volcano plot showing differentially expressed genes in CD69+ B cells between yCRC and oCRC. yCRC versus oCRC, down (up) represents differential genes downregulated (upregulated) in C3_CD69+B cells from yCRC. (**G**) The enriched GO terms in downregulated differential genes of C3_CD69+B cells in yCRC compared with that in oCRC. (**H**) Correlation between abundance of C3_CD69+ B cells and C0_IgG+ plasma cells. Each dot represents one samples (n = 14). P values from spearman correlation test. (**I**) Correlation between C1_CD8_Teff cells interferon gamma pathway activity and C3_CD69+B cells antigen-presentation pathway activity. Each dot represents one samples (n = 14). P values from spearman correlation test. (**J**) Correlation between C1_CD8_Teff cells interferon gamma pathway activity and C3_CD69+B cells Positive regulation of lymphocyte-mediated immunity pathway activity. Each dot represents one samples (n = 14). P values from spearman correlation test.

B cells and plasma cells were the most abundant cell types in our data; we investigated their heterogeneity in yCRC and oCRC by performing a more refined clustering. We extracted a total of 5204 B cells and 27,014 plasma cells. Three B-cell subsets and six plasma cell subsets were identified through marker-based annotation and identification of cluster-specific DEGs (Figure 4A). Since the cluster C7 and C8 express markers of two or more cell types simultaneously, they were considered low-quality cells and were excluded from the subsequent analysis (Figure S4A). In addition to the high expression of B-cell markers such as *MS4A1* and *CD19*, the cluster C3 also highly expressed *CD69*, a marker of tissue-resident memory B cells in lung tissues [33]; we designated it as C3_CD69+B cells (Figure 4A,B). *CD80* is highly expressed in the cluster C6 (Figure 4B); it is a member of the immunoglobulin (Ig) supergene family [34], which is essential for T-cell activation as a T-cell co-stimulatory molecule [35]. *CD80* is generally expressed in activated B cells; naïve resting B cells either express low levels of *CD80* or do not express *CD80* [35,36]. Cluster C6 was defined as C6_CD80+ B cells, which may represent a subset of activated B cells. Two plasma cell subsets highly expressed IgA-related genes. One plasma cell subset with highly expressed IgG-related genes, all of which were annotated according to the classical plasma cell markers (Figure 4B). Cluster C0 and C1 highly expressed immunoglobulin kappa C (*IGKC*), which is associated with good prognosis in a variety of tumors, including CRC, breast cancer, and non-small cell lung cancer [37,38], implying that humoral immunity may also play an essential role in antitumor immunity. In addition, we identified a population of plasma cells characterized by high *CCL5* expression, designated as C5_CCL5+ plasma cells, and a population of C4_IL7R+ plasma cells with high *IL7R* expression (Figure 4B).

We observed a significant enrichment of B cells in yCRC, while plasma cells had a higher abundance in oCRC (Figure 4C and Figure S4B). Plasma cells have a positive effect on tumor immunity [39]. To confirm the prognostic significance of tumor-infiltrating plasma cells in CRC, the gene set of plasma cell markers was defined, and results showed that the survival outcome of patients with a high gene set score in the TCGA CRC cohort was better than that of patients with a low gene set score (Figure 4D). Cell subtypes show the differential distribution in yCRC and oCRC (Figure S4C). C3_CD69+B cells were significantly enriched in yCRC (Figure 4E). To assess whether C3_CD69+B cells have potential functional differences between yCRC and oCRC, we compared the gene expression profiles of the two groups (Figure 4F). Compared with oCRC, C3_CD69+ B cells from yCRC had lower levels of Ig expression including those of *JCHAIN*, *IGLC2*, *IGHG1*, and *IGHA1*. According to the GO analysis, these genes (which were downregulated in the C3_CD69+B cells from yCRC) had immune-activated functions such as antigen processing and presentation and positive regulation of lymphocyte activation (Figure 4G). In addition, the expression levels of the proto-oncogenes *FOS* and *JUNE* were high in yCRC (Figure 4F), and the overexpression of *FOS* affected the isotype switching of B cells [40]. Interestingly, when the relationship between the number of C3_CD69+B cells and plasma cell subtypes in CRC was assessed, we observed that the abundance of C3_CD69+B cells was significantly negatively correlated with the abundance of C0_IgG+ plasma cells (Figure 4H). This finding may imply that the isotype switching of C3_CD69+B cells are more likely to transform to IgG+ plasma cells. B cells also play an essential role in T-cell activation. In the previous analysis of C3_CD69+ B cells, we observed a lower immune activity in the yCRC group; hence, we evaluated the correlation between the activity of the C3_CD69+ B cells antigen-presentation pathway and positive regulation of lymphocyte-mediated immunity pathway activity and the interferon-gamma pathway activity of effector T cells. The immune activity of C3_CD69+ B cells exhibited a significant correlation with the activation of effector T cells (Figure 4I,J). This implies that the downregulated B-cell immunoreactivity in C3_CD69+ B cells from yCRC may also partially contribute to the lower effector T-cell activity in the yCRC.

In summary, yCRC has a higher rate of B-cell infiltration and a lower rate of plasma cell infiltration than oCRC. In addition, we identified a set of plasma cell genes associated with good tumor prognosis. The proportion of C3_CD69+B cells was higher in the yCRC group. However, C3_CD69+B cells from yCRC had lower isotype switching capacity, antigen-presenting activity, and positive regulation of lymphocyte activation activity compared with those from oCRC.

### 3.4. Antitumor Immunity of Myeloid Cells Declines in yCRC

**Figure 5:**
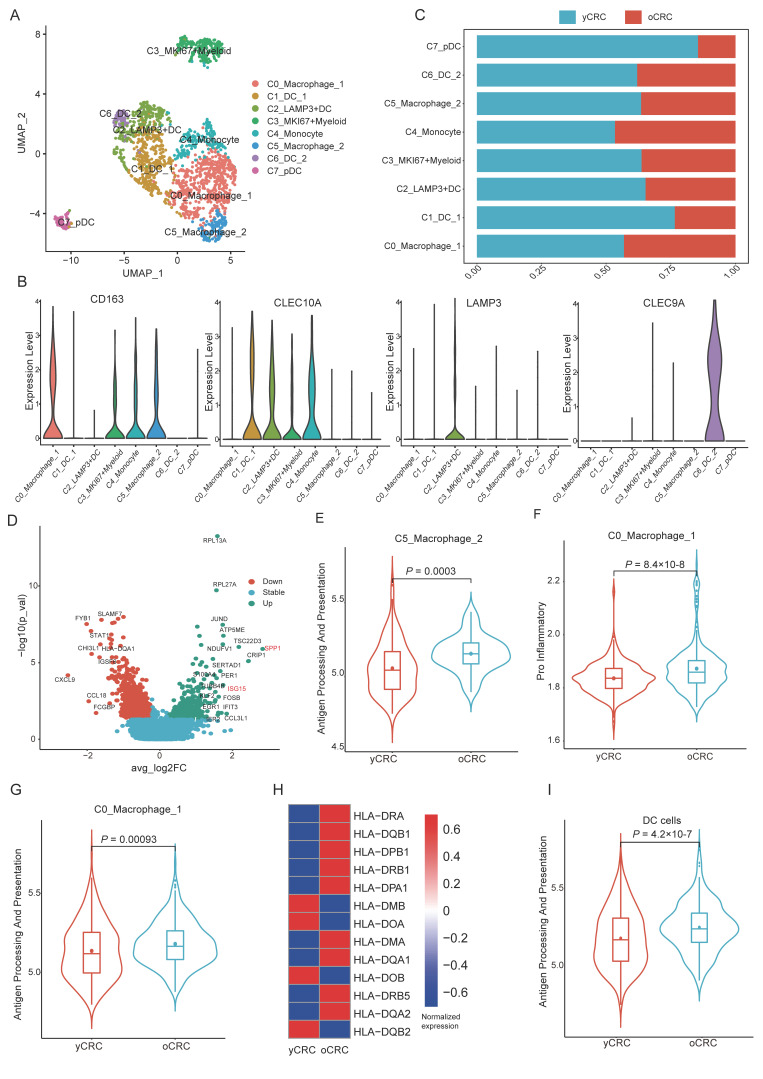
Myeloid cells in yCRC and oCRC. (**A**) UMAP plot showing the subgroups of myeloid cells. (**B**) Violin plot showing the high expression marker genes of myeloid cell subtypes. (**C**) The bar chart showed the proportion of myeloid cell subtypes in yCRC and oCRC. (**D**) Volcano plot showing differentially expressed genes between C5_Macrophage_2 cells from yCRC and oCRC. yCRC versus oCRC, down (up) represents differential genes downregulated (upregulated) in C5_Macrophage_2 cells from yCRC. (**E**) Comparison of Antigen Processing and Presentation activity scores of C5_Macrophage_2 cells between yCRC and oCRC. (**F**) Comparison of Pro-inflammatory activity scores of C0_Macrophage_1 cells between yCRC and oCRC. (**G**) Comparison of Antigen Processing and Presentation activity scores of C0_Macrophage_1 cells between yCRC and oCRC. (**H**) Heatmap showing expression profiles of MHC class II genes in Dendritic cells in different subgroups. (**I**) Comparison of Antigen Processing and Presentation activity scores of Dendritic cells between yCRC and oCRC.

We investigated the heterogeneity of myeloid cells in yCRC and oCRC by performing highly refined clustering. Eight cell subpopulations were identified based on the expression levels of classical markers, including two macrophage subpopulations, one monocyte subpopulation, four dendritic cell (DC) subpopulations, and one myeloid subpopulation in a proliferative state (Figure 5A). Clusters of cells belonging to the same cell type were grouped, and each cluster had a specific gene expression pattern (Figure S5A). Based on the expression levels of markers reported in previous studies, we identified four DC subpopulations (Figure 5A), and C1_DC_1 expressed *CLEC10A*, which was designated as C1_DC_1 (Figure 5B). C2_LAMP3+DC, reported in the literature in recent years, represents a mature DC cell subtype [41] (Figure 5B). C6_DC_2 is characterized as *CLEC9A*+ and is a subtype of cDC1 reported in the literature [25] (Figure 5B and Figure S5A). C7_pDC highly expressed plasmacytoid DCs (pDC) such as *LILRA4* and *GZMB* (Figure S5A). In addition, pDCs were more abundant in the yCRC group (Figure 5C). Macrophages are cells with a high degree of plasticity; we identified two subpopulations of macrophages: C0_Macrophage_1 and C5_Macrophage_2 (Figure 5A). We compared the gene expression profiles of the two macrophage subpopulations and observed that M1-like macrophage markers, including *CCL3*, *CCL4*, and *IL1B*, were highly expressed in C0_Macrophage_1, while *SPP1* and *APOE* genes were highly expressed in C5_Macrophage_2 (Figure S5B). *APOE* was associated with the anti-inflammatory function of macrophages [42]. *SPP1* was a marker of poor prognosis in several types of tumors [43,44] and was significantly associated with M2 polarization of macrophages. Consistent with this finding, the macrophages were assessed by macrophage M1/M2 -related gene sets; C0_Macrophage_1 had a higher M1-like gene score, whereas C5_Macrophage_2 had a higher M2-like gene score (Figure S5C). These results suggest that C0_Macrophage_1 may be similar to M1-like macrophages, while C5_Macrophage_2 is more similar to M2-like macrophages. We performed a GO analysis of the differential genes between the two groups of macrophages. The genes enriched in C0_Macrophage_1 were mainly associated with T-cell activation and lymphocyte activation (Figure S5D); they may play a role in the antigen presentation to T cells to initiate antitumor immune responses.

We compared the gene expression profile of C5_Macrophage_2 in the yCRC and oCRC groups and observed that *SPP1* and *ISG15* gene expression was upregulated in the yCRC group (Figure 5D). *SPP1* plays an essential role in maintaining macrophage M2 polarization, and *ISG15* is associated with poor prognosis in patients with nasopharyngeal carcinoma and induces a macrophage M2-like phenotype [45]. These results imply a more robust anti-inflammatory phenotype of C5_Macrophage_2 in yCRC. To further explore the heterogeneity of macrophage function between the two groups, we evaluated the antigen-presenting and pro-inflammatory functions of macrophages. The antigen-presenting capacity of C5_Macrophage_2 in yCRC was lower than that in oCRC (Figure 5E). Similar results were obtained when the function of C0_Macrophage_1 in relation to antitumor immunity was evaluated. The results showed that the pro-inflammatory functions and antigen-presenting of C0_Macrophage_1 were weaker in yCRC compared with that in oCRC (Figure 5F,G).

DCs are the most potent antigen-presenting cells, activating primitive T cells, and recent data highlight the vital role of DC cells in antitumor immunity [46,47]. We assessed the expression levels of MHC class II genes that may indicate the antigen-presenting ability in yCRC and oCRC (Figure 5H). The MHC class II gene expression in DCs was lower in yCRC compared with that in oCRC. Similarly, the scores of antigen processing and presentation pathways in yCRC were lower than that in oCRC (Figure 5I).

We next focused the interactions between myeloid cells and T cells, with myeloid cells and T cells showing extensive cellular interactions (Figure S5E). C5_Macrophage_2 established strong connections with themselves and other cells through the *SPP1* signaling (Figure S5F). Previous studies [48] have shown that *SPP1* inhibits T-cell activation by binding CD44 and contributes to immune tolerance in CRC; therefore, targeting *SPP1* signaling is a promising therapeutic option for CRC, especially in patients of yCRC.

Two sets of macrophage subtypes with different polarization statuses were identified in the yCRC and oCRC groups. In the yCRC group, C5_Macrophage_2 highly expressed *SPP1*, and *ISG15*, associated with macrophage M2 polarization, had more robust anti-inflammatory properties. Moreover, the antigen-presenting function of C5_Macrophage_2 was lower in yCRC compared with that in oCRC. In addition, the pro-inflammatory and antigen-presenting functions of C0_Macrophage_1 were lower in yCRC. The antigen-presenting capacity of DCs was diminished in yCRC. By constructing the interaction networks between myeloid and T cells, we suggested that targeting the *SPP1* signaling pathway may be a more promising therapeutic option for yCRC.

### 3.5. Heterogeneity of Malignant Cells and Interactions with Immune Cells in the TME

We extracted and evaluated 23,751 epithelial cells. CNV analysis was used to distinguish malignant epithelial cells from normal epithelial cells. We used inferCNV to calculate the CNV of epithelial cells from single-cell transcriptome profiles (Figure S6A). Epithelial cells with high CNV were classified as malignant cells, and a total of 17,833 malignant cells were identified (Figure S6B). We investigated whether malignant cells from different subgroups have different gene expression patterns. Therefore, GSVA was performed on cells from the yCRC and oCRC groups. The GSVA results indicate the enrichment of the IL-6/JAK/STAT3 pathway, epithelial-mesenchymal transition pathway, and hypoxia pathway in the yCRC group (Figure S6C). In previous studies, the IL-6/JAK/STAT3 pathway in CRC promotes tumor genesis and tumor growth, as well as inhibits tumor cell apoptosis [49,50]. In line with this finding, we found that malignant cells in the yCRC group showed lower apoptosis activity (Figure S6D). Epithelial-interstitial transition pathways and hypoxic pathways have been reported in a variety of tumors and are associated with poor tumor prognosis [51,52]. In the oCRC group, the MYC pathway, involved in cell growth, apoptosis, and metabolism, is significantly upregulated. A recent study revealed age-related CRC proteomic features, and this finding is consistent with the discovery of highly expressed MYC in oCRC, thus suggesting that proteins targeting the MYC genome may be a better choice for treatment of oCRC patients [53].

**Figure 6:**
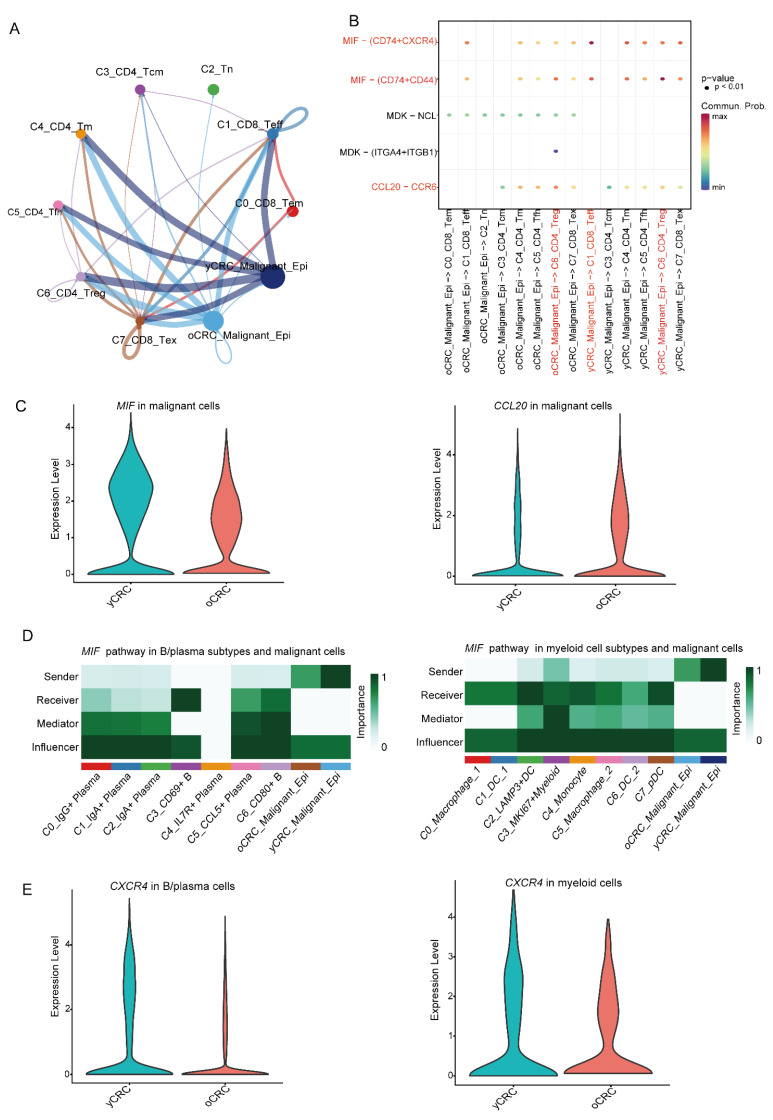
Heterogeneity of malignant cells and interactions with immune cells in the TME. (**A**) The strength network of inferred cellular interactions among T cell subtypes and malignant cells. (**B**) Ligand-receptor interaction probabilities of among T cell subtypes and malignant cells. (**C**) Violin plot showing MIF (left) and CCL20 (right) gene expression in malignant cells of yCRC and oCRC. (**D**) Heatmap shows the relative importance of each cell subtypes based on the computed four network centrality measures of MIF signaling network among cell subtypes and malignant cells. B/plasma subtypes and malignant cells (left). myeloid cell subtypes and malignant cells (right). (**E**) Violin plot showing CXCR4 gene expression in B/plasma cells (left) and myeloid cells (right) of yCRC and oCRC.

The TME is a complex ecosystem where cell-to-cell crosstalk determines the biology of the tumor and its response to treatment. To systematically characterize the interaction of cells in the TME of yCRC and oCRC, the R package CellChat was used to explore the crosstalk between malignant cells and major immune cells. We evaluated the crosstalk between T cells and malignant cells and observed that the *MIF* signaling pathway showed extensive intercellular communication (Figure 6A,B). We compared the expression levels of the ligand *MIF* in the yCRC group with those in the oCRC group and observed that *MIF* showed higher expression in the yCRC group (Figure 6C). The *CCL20*-*CCR6* pathway shows the strongest communication between C6_CD4_Treg cells and oCRC_Malignant_Epi (Figure 6B), consistent with a previous suggestion that *CCL20*-*CCR6* is associated with Treg function suppression [54] Ligand *CCL20* showed higher expression in the oCRC group (Figure 6C). Surprisingly, when the crosstalk between malignant cells, B cells, plasma cells, and myeloid cells was evaluated, the *MIF* signaling pathway also showed extensive intercellular crosstalk (Figure 6D). Similarly, *CXCR4*, the receptor for the *MIF* signaling pathway, was expressed in a much higher level in B/plasma cells and myeloid cells in the yCRC group (Figure 6E). *MIF* is associated with a poor prognosis, as a pro-inflammatory cytokine whose expression and secretion levels are elevated in most types of solid tumors [55]. *MIF* contributes to immune evasion and immunosuppression in the TME by regulating signals in immune cells [56]. Tumor-derived *MIF* has an inhibitory effect on the activation and proliferation of T cells. In mononuclear macrophages, tumor-derived *MIF* can initiate monocyte-dependent angiogenesis, suggesting an important functional role of *MIF* in the polarization of M2 macrophages. *MIF* promotes B-cell migration through the synergy of *CXCR4*, which may contribute to the recruitment of B cells in tumors.

In summary, the epithelial cells in yCRC and oCRC are highly heterogeneous. Moreover, there is extensive cellular crosstalk between malignant cells and immune cells in the TME, and this intercellular crosstalk may contribute to the development of a suppressive tumor immune microenvironment.

## 4. Discussion

In this study, we depicted an outline of the TME associated with age in CRC and revealed significant differences in the TME between yCRC and oCRC. B cells and naïve T cells were enriched in the TME of yCRC, whereas plasma cells and effector T cells, which were associated with anti-tumor immunity, were enriched in the TME of oCRC. The TME was heterogeneous between the two groups, with yCRC having a more suppressive TME compared with the oCRC. These findings may provide valuable references for further research on the biological features of CRC and the development of new therapeutic targets.

T cells manifested heterogeneity between yCRC and oCRC. The proportion of CD8+ effector T cells was significantly lower in yCRC compared with that in oCRC. GSVA between the two groups demonstrated that effector T cells may be in a more resting state in yCRC, with lower effector activity and proliferative activity compared with that in oCRC. We found lower antigen presentation and lymphocyte activation in C3_CD69+ B cells in the yCRC group compared with that in the oCRC group, which may also lead to lower effector T-cell activity in the yCRC. In addition, C5_Macrophage_2 has a stronger *SPP1* signaling pathway interacting with effector T cells in yCRC, which further inhibits the effector T cell activity. Consistently, a recent study showed that CD8+ effector T-cell infiltration was lower in young mice compared with that in older mice and showed lower antitumor capacity in young mice [57]. By contrast, the yCRC had a higher proportion of naïve T cells. Previous studies reported that thymic degeneration with aging may lead to a decrease in the number of naïve T cells [58]. CD4+ T cell trajectory analysis showed that naïve T cells have a differentiation trajectory toward Treg cells, which are mainly located at the end of the CD4+ T cell differentiation trajectory. In our data, the proportion of Treg cells, which have immunosuppressive functions, was not significantly different between the two groups. However, Treg cells in yCRC express higher levels of co-stimulatory genes (*TNFRSF4*/*TNFRSF18*) and immune checkpoint molecules (*CTLA4*) compared with that in oCRC. Co-stimulatory receptors play a key role in Treg differentiation and functions [59]. *CTLA4*, the co-inhibitory receptor on Treg cells, inhibits glycolysis in T cells, which promotes Treg cells homeostasis [60]. The overexpression of co-inhibitory receptors induced by Treg cells is one of the prominent mechanisms by which they suppress antitumor immunity [61]. Cellular metabolism is closely related to the function and stability of Treg cells [62]. Oxidative phosphorylation and mitochondrial metabolism play key roles in the immunosuppressive activity of Treg cells [63]. Treg cells in yCRC have higher oxidative phosphorylation metabolic activity compared with that in oCRC, which may indicate a higher immunosuppressive activity of Treg cells in yCRC. Consistently, the suppressive activity of Treg cells decreases with increasing age [64]. Our data also emphasized that the crosstalk between other cells in the TME and Treg cells can also affect their immunosuppressive activity.

Further analysis of B cells and plasma cells revealed that C3_CD69+ B cells were predominantly expressed in yCRC. A previous study [65] reported a decreased rate of B cell generation in older adults, which possibly contributed to the lower proportion of B cells observed in oCRC. In addition, the C3_CD69+B cells in yCRC were less likely to be isotype switching compared with those in oCRC. The overexpression of the proto-oncogene *FOS* interferes with the Ig class-switching recombination mechanism of B cells [40]. The C3_CD69+B cells in yCRC had higher levels of *FOS* expression, which may be the reason for the isotype-switching differences between the two groups of C3_CD69+B. Moreover, the abundance of C3_CD69+B cells showed a significant negative correlation with the abundance of C0_IgG+ plasma cells; hence, we speculate that the isotype switching of C3_CD69+B cells are more likely to transform to IgG+ plasma cells. By contrast, the C3_CD69+B cells in yCRC have a weaker capacity for isotype switching compared with that in oCRC, which may also contribute to the enrichment of C3_CD69+B cells in yCRC. The GO analysis of differential genes between the two groups showed that the C3_CD69+B cell antigen processing and presentation and positive regulation of lymphocyte activation were downregulated in the yCRC group.

In myeloid lineage, DC cells are the main antigen-presenting cells. In yCRC, DC cells have lower expression levels of MHC Ⅱ genes and lower scores of the antigen processing and presentation pathway. DC cells are innate immune antigen-presenting cells, and aging has a much less pronounced effect on innate immunity than on adaptive immunity. The ability of DC cells to activate antigen-specific T cells is preserved with aging; however, it has been reported that aging impairs the initiation of T cells by DC cells [66,67]. Therefore, the differences across model systems remain controversial and require further investigation. pDCs expressed in several types of tumors are associated with poor prognosis and can impair antitumor immunity [68,69]. In this study, the pDCs were predominantly derived from patients with yCRC, which may also lead to a more suppressive immune microenvironment. Plasticity and functional polarization are characteristics of macrophages in the TME. To further reveal the functional status of different macrophage subpopulations, we assessed the scores of the classical M1-like and M2-like macrophage gene sets. Two groups of macrophages with opposite polarization statuses were identified, both of which did not show significant differences in proportion between the yCRC and oCRC groups. However, genes associated with M2 polarization (*SPP1* and *ISG15*) were more highly expressed in yCRC C5_Macrophage_2, possibly indicating that M2-like macrophages in yCRC have a stronger anti-inflammatory property. After further evaluating the macrophage function, we observed that the antigen-presenting functional activity of C5_Macrophage_2 and C0_Macrophage_1 was lower in yCRC compared with that in oCRC. In addition, the pro-inflammatory function of C0_Macrophage_1 was also lower in yCRC. In yCRC, M2-like macrophages have a stronger anti-inflammatory function; in oCRC, M1-like macrophages have a stronger pro-inflammatory function. In the inference of cellular interactions between myeloid and T cells, we found a widespread presence of the *SPP1* signaling pathway in C5_Macrophage_2. Moreover, the expression of *SPP1* was higher in the young group, which may imply that the effector T-cell activity function was suppressed by the *SPP1* pathway in the yCRC group. This may also help explain the resting state of effector T cells in yCRC.

Malignant cells show strong heterogeneity in the TME. We revealed two groups of malignant cell types derived from yCRC and oCRC, respectively, which have completely different transcriptional profiles. Malignant cells in yCRC had a lower apoptotic activity compared with that in oCRC, which may correlate with the more aggressive nature of yCRC (Figure S6D). Furthermore, malignant cells may be associated with the induction of an immunosuppressive microenvironment in CRC. Intercellular interaction analysis was performed based on ligand-receptor pairs to explore the crosstalk between malignant cells and immune cells in the TME. We observed the universality and importance of the *MIF* pathway in communication between malignant cells and immune cells. In particular, *MIF* showed higher expression in yCRC, which implies stronger cellular interactions of the *MIF* pathway. *MIF* is secreted by malignant cells and has a suppressive effect on the activation and proliferation of effector T cells [70]; this finding indicates that the effector T cells from yCRC had lower proliferative activity and cytotoxicity compared with those from oCRC. *MIF*-*CXCR4* contributes to the migration of B cells [71], while *CXCR4* is more highly expressed in the yCRC group of B cells. Thus, the stronger *MIF*-*CXCR4* pathway interactions in yCRC may help explain the enrichment of B cells in yCRC. *MIF* plays an important role in the polarization of M2 macrophages [72,73], which may partially explain the stronger anti-inflammatory effect of M2-like macrophages in yCRC. In addition, the oCRC_Malignant_Epi may inhibit the immunosuppressive function of Treg cells via *CCL20*-*CCR6* interaction. In summary, malignant cells play an important role in the formation of a suppressive immune microenvironment in CRC, and blocking the *MIF* pathway may be an effective target for CRC therapy, especially in yCRC.

In conclusion, our study revealed significant TME heterogeneity at the single-cell level in yCRC and oCRC. yCRC had a more suppressive immune microenvironment compared with oCRC. Our results may serve as a basis for developing appropriate treatments for patients with CRC.

## Data Availability

The data that support the findings of this study are available from the corresponding author on reasonable request.

## Supplementary Materials

The following supporting information can be downloaded at: https://www.mdpi.com/article/10.3390/biom12121860/s1, Figure S1: Cell markers and cell proportions of different cell types; Figure S2: Marker genes in each T cell subtype; Figure S3: Comparison of proliferation activity scores of C1_CD8_Teff cells; Figure S4: Cell markers and cell proportions of B cells and plasma cell subtypes; Figure S5: Polarization of macrophages in the TME; Figure S6: Heterogeneity of malignant cells; Table S1: Clinical and pathological characteristics of the patients.
