# Supplementary material for: Single-Cell RNA Sequencing Reveals Heterogeneity in the Tumor Microenvironment between Young-Onset and Old-Onset Colorectal Cancer
Source: Biomolecules. 2022 Dec 12;12(12):1860. doi: 10.3390/biom12121860 (PMC9776336; doi:10.3390/biom12121860)
Supplement: Supplementary file 1 [file biomolecules-12-01860-s001.zip › Supplementary Figure Legends20221203.pdf]

Supplementary Table S1

Clinical and pathological characteristics of the patients.

Supplementary Figure Legends

Figure S1

(A) The UMAP plot showing the cells of 23 cell clusters.

(B) Violin plots displaying the distribution of expression of known marker genes across diverse cell types.

(C) Abundance ratios between yCRC and oCRC in each cell types (estimated by Wilcoxon test).

Figure S2

(A) Heatmap showing the marker genes in each T cell subtype.

Figure S3

(A) Comparison of cell proliferation activity scores of C1_CD8_Teff cells between yCRC and oCRC.

Figure S4

(A) Heatmap showing the marker genes in each cell subtype from B cells and plasma cells.

(B) Abundance ratios of Plasma cells between yCRC and oCRC (estimated by Wilcoxon test).

(C) The bar chart showing the proportion of cell types in yCRC and oCRC.

Figure S5

(A) Heatmap showing differential expression of genes in each myeloid cell subtype.

(B) Volcano plot showing differentially expressed genes between C0_Macrophage_1 and C5_Macrophage_2.

(C) Comparison of GSVA scores of M1 polarization and M2 polarization between C0_Macrophage_1 and C5_Macrophage_2. Significance levels are indicated as $^{**}P<0.01$ and $^{***}P<0.001$.

(D) The enriched GO terms in upregulated differential genes of C0_Macrophage_1 versus C5_Macrophage_2.

(E) The strength network of inferred cellular interactions among T cell subtypes and myeloid cell subtypes.

(F) Cellular interactions of SPP1 signaling pathway.

Figure S6

(A) Heatmap showing the baseline expression for genes in stromal cells (top). The heatmap of the relative expression density of genes in each chromosome by

comparing the epithelial cell genome with stromal cell reference genomes.(bottom)

(B) The UMAP plot showing the cells colored by different inferCNV result.

(C) Differences in Hallmark pathways activities scored per cell by GSVA between yCRC and oCRC malignant cells. t values are from linear models, corrected for effects from the patient of origin.

(D) Comparison of cell apoptosis activity scores of malignant cells between yCRC and oCRC. Significance level is indicated as ***P<0.001.
