# Supplementary figures and images for: Single-Cell RNA Sequencing Reveals Heterogeneity in the Tumor Microenvironment between Young-Onset and Old-Onset Colorectal Cancer
Source: Biomolecules. 2022 Dec 12;12(12):1860. doi: 10.3390/biom12121860 (PMC9776336; doi:10.3390/biom12121860)

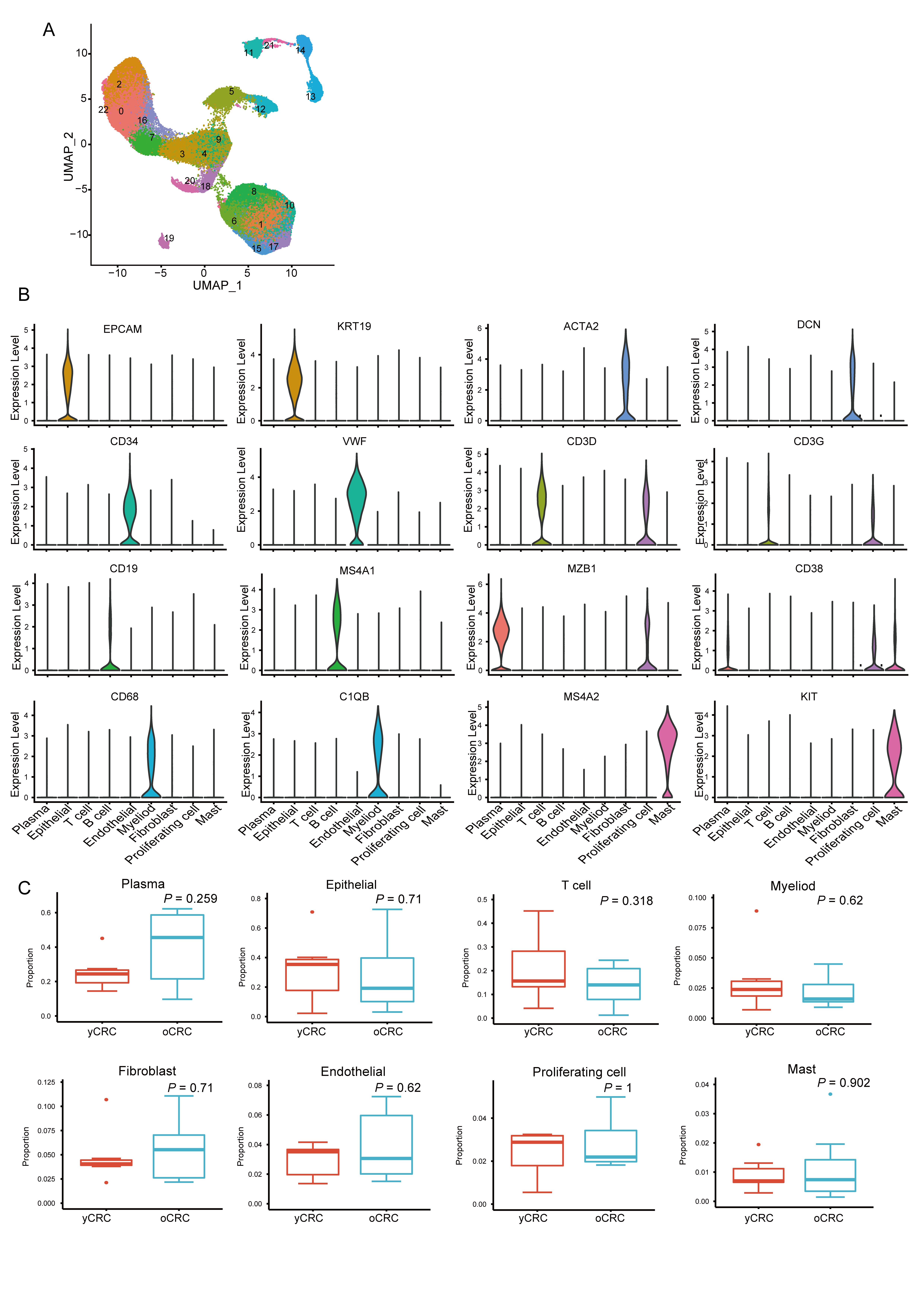

Supplement: Supplementary file 1 [file biomolecules-12-01860-s001.zip › FigureS1.tif]

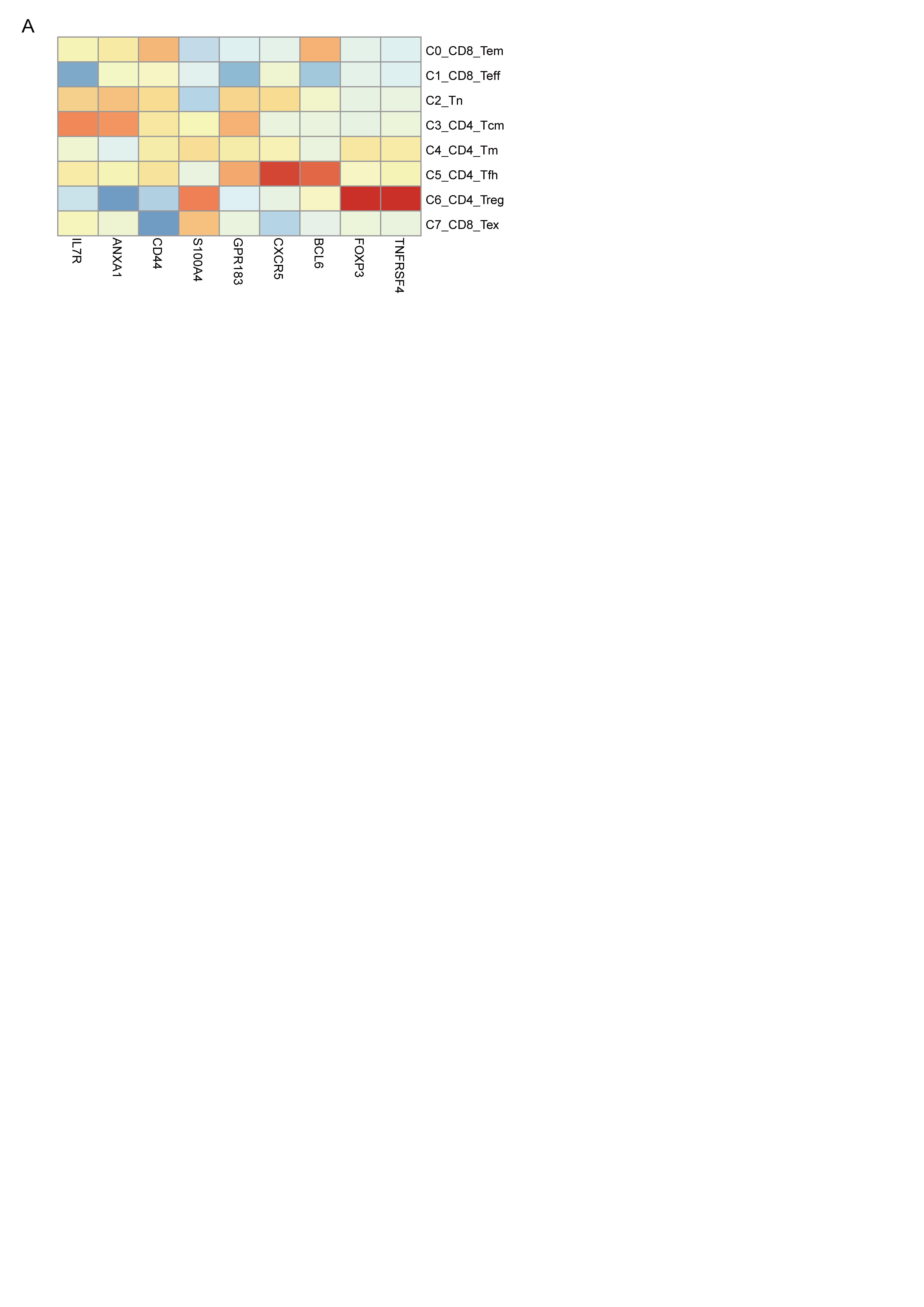

Supplement: Supplementary file 1 [file biomolecules-12-01860-s001.zip › FigureS2.tif]

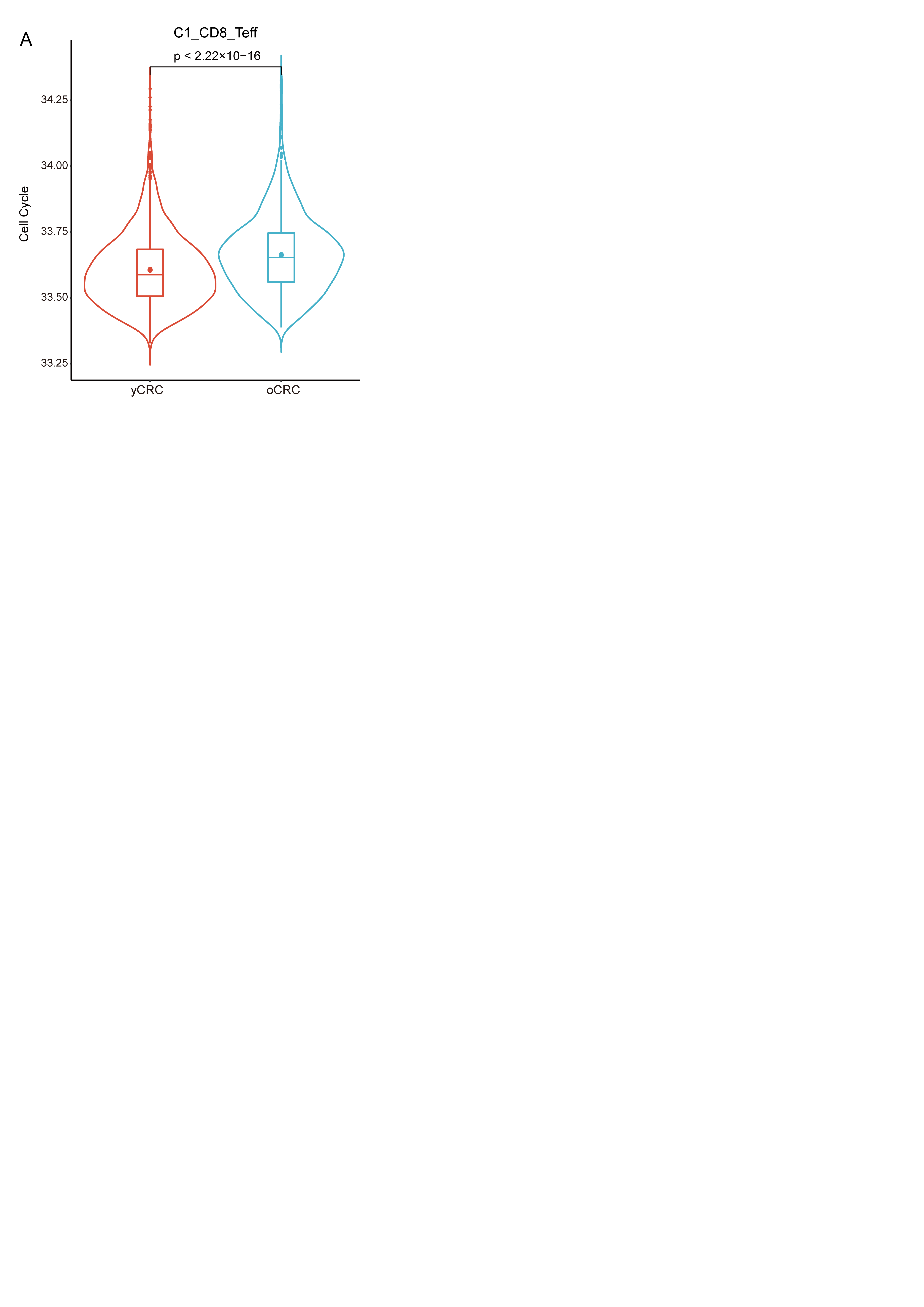

Supplement: Supplementary file 1 [file biomolecules-12-01860-s001.zip › FigureS3.tif]

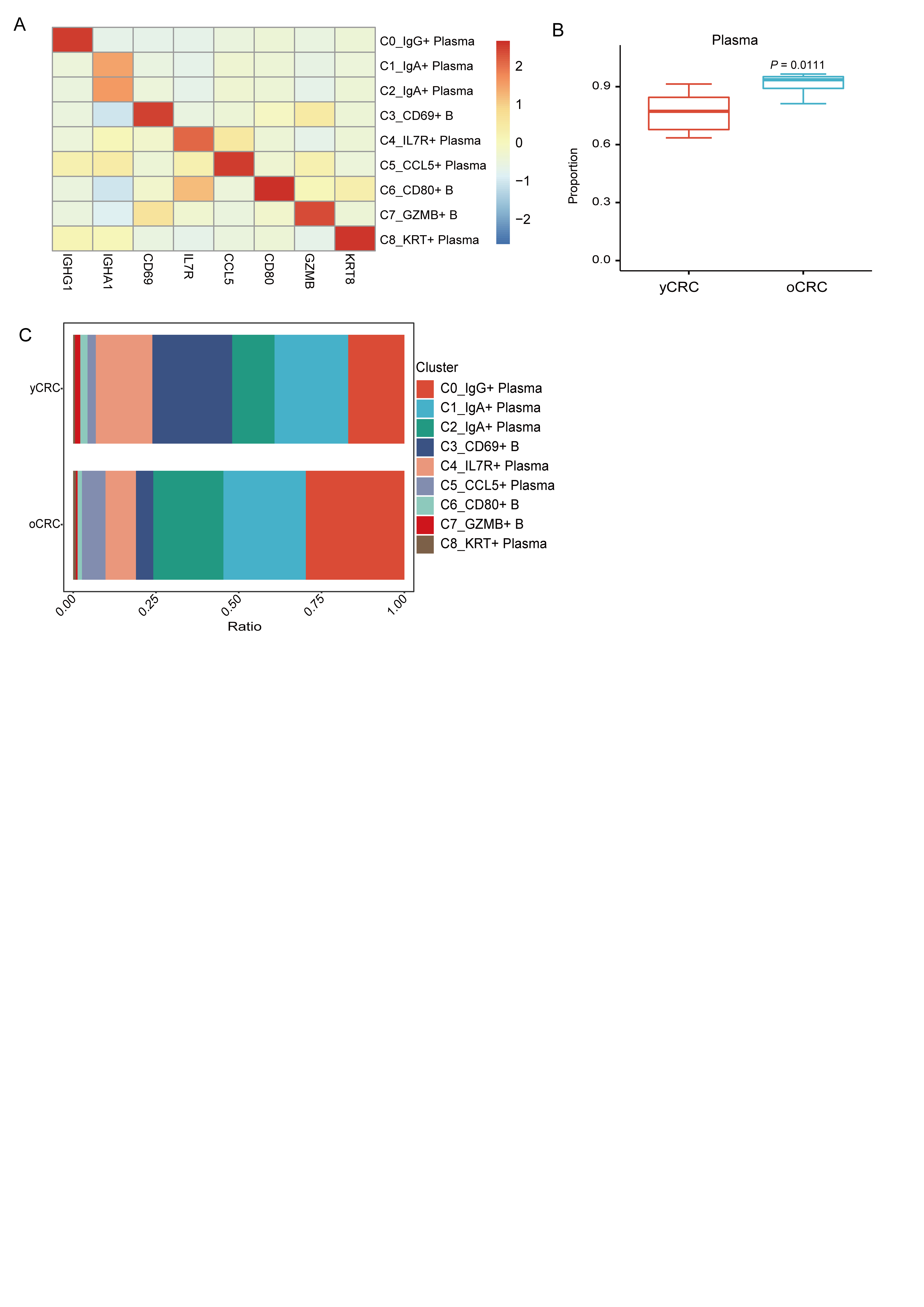

Supplement: Supplementary file 1 [file biomolecules-12-01860-s001.zip › FigureS4.tif]

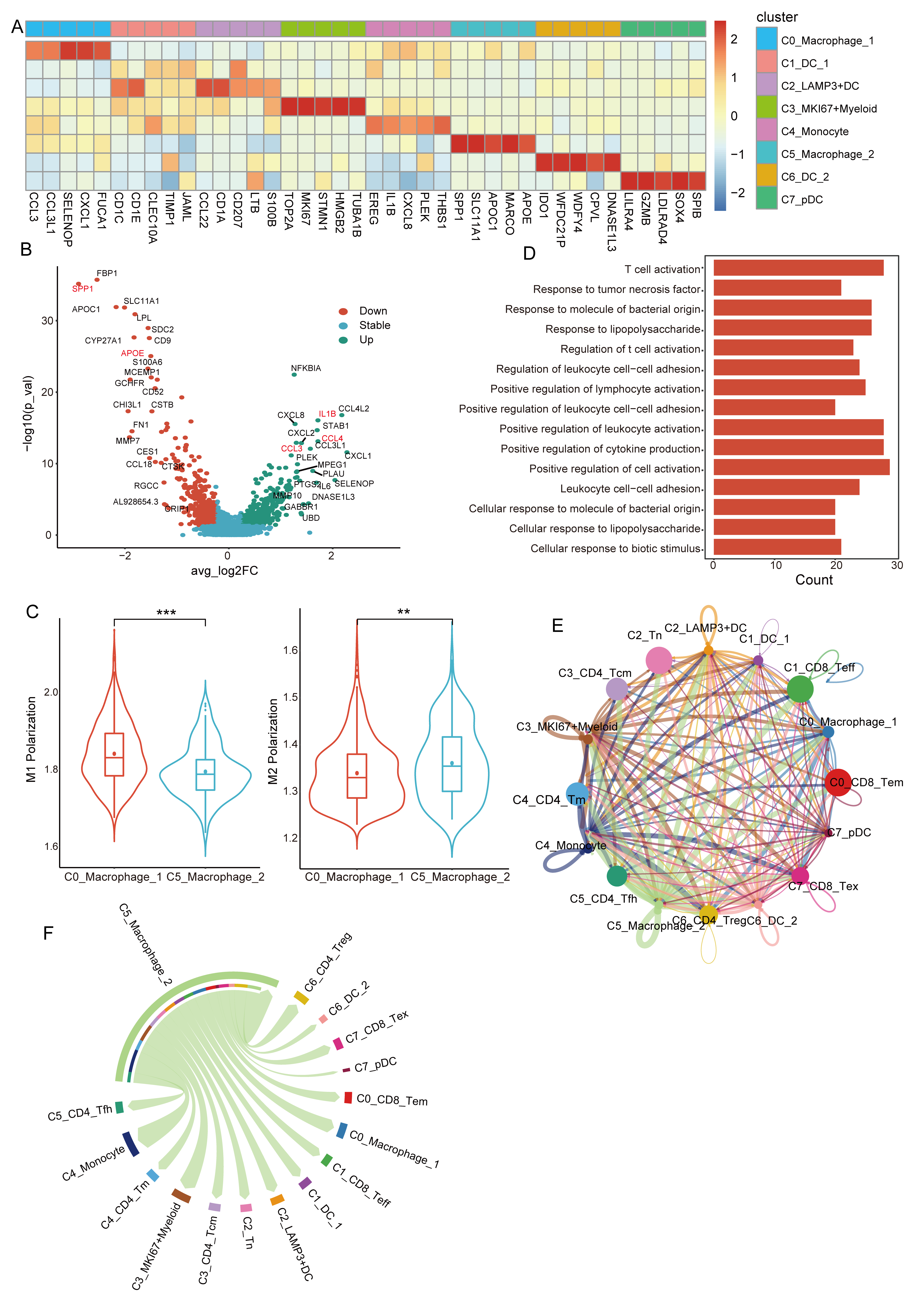

Supplement: Supplementary file 1 [file biomolecules-12-01860-s001.zip › FigureS5.tif]

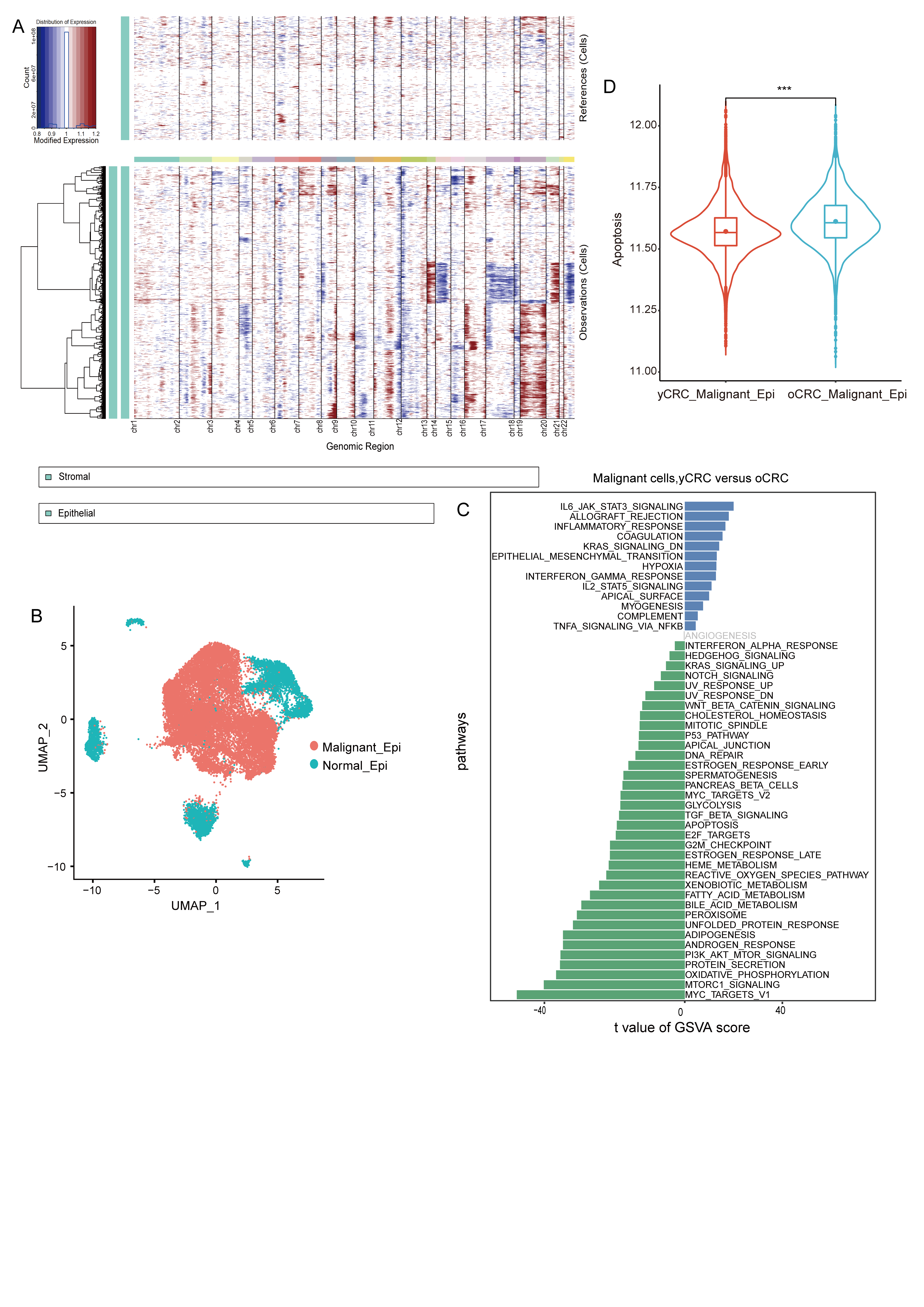

Supplement: Supplementary file 1 [file biomolecules-12-01860-s001.zip › FigureS6.tif]
